# A Novel Panel of Serum Biomarkers for MPM Diagnosis

**DOI:** 10.1155/2017/3510984

**Published:** 2017-02-28

**Authors:** A. Bonotti, R. Foddis, S. Landi, O. Melaiu, C. De Santi, L. Giusti, E. Donadio, F. Ciregia, M. R. Mazzoni, A. Lucacchini, M. Bovenzi, M. Comar, E. Pantani, A. Pistelli, A. Cristaudo

**Affiliations:** ^1^Operative Unit of Occupational and Environmental Medicine, University Hospital of Pisa, Pisa, Italy; ^2^Department of Translational Research and of New Technologies in Medicine and Surgery, University of Pisa, Pisa, Italy; ^3^Department of Biology, University of Pisa, Pisa, Italy; ^4^Department of Pharmacy, University of Pisa, Pisa, Italy; ^5^Department of Medical Sciences, University of Trieste, Trieste, Italy

## Abstract

Exposure to asbestos is the main cause of malignant pleural mesothelioma (MPM), a highly aggressive cancer of the pleura. Since the only tools for early detection are based on radiological tests, some authors focused on serum markers (i.e., mesothelin). The aim of this study was the evaluation of new serum biomarkers to be used individually or in combination, in order to improve the outcome of patients whose disease would be diagnosed at an earlier stage. Serum and plasma were available from 43 subjects previously exposed to asbestos and 27 MPM patients, all being epithelioid type. All the new markers found differentially expressed in MPM and healthy subjects, by proteomic and genomic approaches, have been validated in the serum by the use of specific ELISA. The combined approach, using tools of genomics and proteomics, is found to be highly innovative for this type of disease and led to the identification of new serum markers in the diagnosis of MPM. These results, if confirmed in a larger series, may have a strong impact in this area, because early detection of this cancer in people at high risk could significantly improve the course of the disease and the clinical approach to an individualized therapy.

## 1. Introduction

Malignant mesothelioma is a highly aggressive cancer, unresponsive to chemotherapy, radiotherapy, or surgical resection. In particular, Malignant Pleural Mesothelioma (MPM) without treatment is associated with a poor prognosis, with a median survival ranging from 4 to 10 months [1], but tumor stage, histology, and type of therapy had a significant effects on survival [2]. MPM represents less than 1% of known cancers but its incidence will continue to increase significantly, at least until 2019-2020 [1]. This uninterrupted increase in MPM is consistently attributable to the inhalation of asbestos fibres. Widespread employment of asbestos in the last century [3], combined with the high biopersistence of asbestos fibres, accounts for the extraordinary proportion of people exposed to asbestos for occupational or environmental reasons. In fact, about 20–40% of adult men report a work history that may have entailed asbestos exposure on the job (Helsinki criteria, 1997).

At this time the only instruments for screening and early diagnosis are based on radiological tests with evident ethical and economic problems. For this reason, some authors are evaluating biological indicators with the significance of screening and early diagnosis markers, such as serum and plasmatic osteopontin (OPN) and serum mesothelin-related proteins (SMRP) [4–8]. However, the clinical limitation of these studies is that SMRP and pOPN lack sensitivity and specificity, respectively, limiting their usefulness for diagnosis and disease therapy monitoring of patients. So, several researches are evaluating combination of biomarkers for earlier and better diagnosis of MPM cases.

Very recently, we published a paper on gene expression in MPM, suggesting TIMP3, THBS2, and MLSN as tumor genes in MPM [9]. In 2014, we also reported a proteomic research, where we highlighted Desmin, Vimentin, and Pre-Lamin A/C as promising markers for MPM [10].

In the present study, we aim to validate all these markers founded in our recent studies in serum, alone or in combination with other interesting markers, in order to identify a panel of biomarkers useful in the surveillance of high-risk people.

## 2. Materials and Methods

### 2.1. Patients

Serum and plasma were available from 43 subjects previously exposed to asbestos (controls), not affected by MPM or another neoplasm, presenting at the University Hospital of Pisa in years 2008–2013, within a follow-up program of health surveillance established by the Occupational Medicine Unit.

The program consists of the following: at the initial assessment a complete medical examination, including chest X-ray, spirometry, and DLCO, and analysis of mesothelin and osteopontin (ELISA) is carried out.

27 MPM patients were enrolled at the time of diagnosis, before beginning any therapeutic treatment. All MPMs were epithelioid types, histologically confirmed.

Each sample was coded at the moment of collection, in order to avoid any personal identification: all data were treated in accordance with the Italian law of privacy ( number 675/96) and handled as approved by the Ethical Board and the Helsinki declaration.

For all patients, age, sex, smoking habit, years of work, and asbestos exposure were indicated. For MPM group, the average number of asbestos exposure was computed for only the one who was exposed.

### 2.2. ELISA Analysis

The Human Osteopontin Assay Kit (IBL, Gunma, Japan), a commercially available ELISA (enzyme-linked immunosorbent assay), was used to determine the level of pOPN. Briefly, plasma samples were diluted 1 : 10 with EIA buffer. Blank, standards, and samples were applied in duplicate in a O-17 antibody precoated microwell plate and were incubated for 1 hour at 37°C. The plate was washed eight times and 100_l of labeled antibody 10A16 was added in each well. After an incubation period of 30 minutes at 4°C, the plate was washed nine times and chromogen was added. The plate was incubated for 30 minutes at room temperature in the dark and stop solution was added. Absorbance read at 450 nm was used to quantify the OPN concentration in ng/mL by comparison with the standard curve plotted by Microsoft Excel.

Serum mesothelin concentration was measured using a sandwich-type ELISA, Mesomark (Cisbio International, Gif/Yvette, France), according to instructions [11]. Briefly, patient serum samples were diluted 1 : 101 with the assay diluent. Next, 100_l of blank, provided standards, and samples were applied in duplicate in a microwell plate precoated with antibody 4H3. After 1-hour incubation on a shaking plate at room temperature, the wells were washed and antibody OV569-HRP was added for 1 hour. After a second washing step, TMB substrate was added to wells for 15 minutes, and then 100_l of stop solution was added. Absorbance read at 450 nm was used to quantify the SMRP concentration in nM by comparison of mean of the duplicate measurement with a calibration curve fitted by CourbesRD software (InstallShield Corporation, Inc., France).

Cytokines and grow factors were measured using magnetic bead multiplex immunoassays (Bio-Plex, BIO-RAD Laboratories, Milano, Italy). Luminex multiplex panel technology was used for simultaneous measurement of a panel of the following analytes: IL6, TNF-a, IL-5, Eotaxin, FGF-basic, PDGF-bb, VEGF, IP-10, CTACK, HGF, and SCGF-b. Briefly, 50 mL of diluted (1 : 4) serum samples and reaction standards were added, in duplicate, to a 96 multiwells plate containing analyte beads followed by incubation for 30 minutes at room temperature. After washing, the antibody-biotin reporter was added and incubated for 10 minutes with streptavidin phycoerythrin. The levels of the cytokines were determined using the Bio-Plex array reader (Luminex, Austin, TX). The Bio-Plex Manager software optimized the standard curves automatically and returned the reading data as Median Fluorescence Intensity (MFI) and concentration (pg/mL).

Vimentin ELISA kit (MyBioSource, San Diego, California, USA, cat. MBS721933), Desmin ELISA kit (USCN, Business Co., Ltd.), THBS2 ELISA kit by MyBioSource (San Diego, California, USA, cat. MBS175793), Fibulin 3 ELISA kit by USCN (Life Science Inc., cat. E95422Hu), and TIMP3 ELISA kit by Abcam (cat. Ab119608) were used for quantitative detection of human Vimentin, Desmin, Thrombospondin, Fibulin, and Timp3 in serum, respectively, according to manufacturer's instructions. Briefly, microtiter plates provided in these kit had been precoated with specific antibody. Standards and samples were then added to the appropriate microtiter plate wells with a biotin-conjugated specific antibody. After incubation, wells were washed and then incubated with HRP enzyme substrate. The reactions were stopped and the intensity of color was measured at 450 nm in a microplate reader.

The prelamin A/C concentration in serum samples was determined by home-made ELISA kit. MaxiSorp 96 microtiter plates (Nunc, GmbH, Germany) were coated with 100 *μ*L of prelamin (2.5 *μ*g/mL) (Recombinant Human Prelamin A (number REP0039, Diatheva srl, Fano, Italy) prepared in carbonate bicarbonate buffer (pH 9.75) and incubated for 48 hours at room temperature. The coated wells were blocked with phosphate buffered saline (PBS) containing 3% bovine serum albumin (BSA, Sigma) for two hours at room temperature (RT). Plates were then washed with PBS containing 0.1% Tween-20. Welles were incubated with 250 ng of primary antibody preLMN A/C (Rabbit Anti-Human Prelamin A (number ANT0045, Diatheva srl, Fano, Italy) in the presence of serial dilutions preLMN A/C (25–800 ng) or serum samples for 2 hours at 37°C. After the incubation period the wells were decanted, washed five times, and incubated with 1 : 25000 of the anti-rabbit secondary antibody, HRP conjugated for 1 hour at 37°C. Plates were then washed and incubated with tetramethylbenzidine (TMB), as the substrate, for 10 minutes at RT. Optical densities (OD) were measured at 450 nm using a 96-well plate reader ELISA spectrophotometer (Wallac Victor2 1420 multilabel counter PerkinELMER).

### 2.3. Statistical Analysis

All markers were analyzed to define the potential Gaussian distribution, in order to choose the appropriate statistical method, using Kolmogorov-Smirnov test. Differences between groups were analyzed by Mann–Whitney test, since the variables were not normally distributed, and all values were shown as median, 25th, and 75th percentiles. On the other hand, Gaussian variables were analyzed using Student's* t*-test for unpaired samples and reported as mean plus or minor standard deviation (SD).

Logistic regression was used to determine the weight given to each marker and then to calculate a specific formula to provide a combined risk index. In order to estimate whether this marker combination might increase the markers performance in MPM detection, receiver-operating characteristic (ROC) curves were plotted and the areas under curves (AUC) were calculated with their 95% confidence intervals (95% CI) using standard techniques to evaluate sensitivity and specificity of each marker and their combination. The Youden Index (1 + Sensitivity − (1 − Specificity)) was used to assess the best cut-off for each marker or marker combination. The best cut-off was defined as the better combination of sensitivity and specificity. The index gives equal weight to false positive and false negative values, so all tests with the same value of the index give the same proportion of total misclassified results. Statistical analysis was performed with SPSS v20.0 (Statistical Package for the Social Sciences).

## 3. Results

Demographic, smoking habit, and working history data of people under investigation are shown in Table 1.

Only Eotaxin, VEGF, HGF, Lamin, and Vimentin, since normally distributed, were reported as mean, standard deviation and minimum and maximum values. These markers were analyzed with a Student's* t*-test. On the contrary, the other markers were showed as median, 25° percentile, and 75° percentile, due to their non-Gaussian distribution, and they were analyzed with nonparametric statistical method (Mann–Whitney test). All data are shown in Table 2.

All markers differentially expressed in workers previously exposed to asbestos and MPM cases in statistical analysis were evaluated with ROC curve (Table 3).

**Figure 1:**
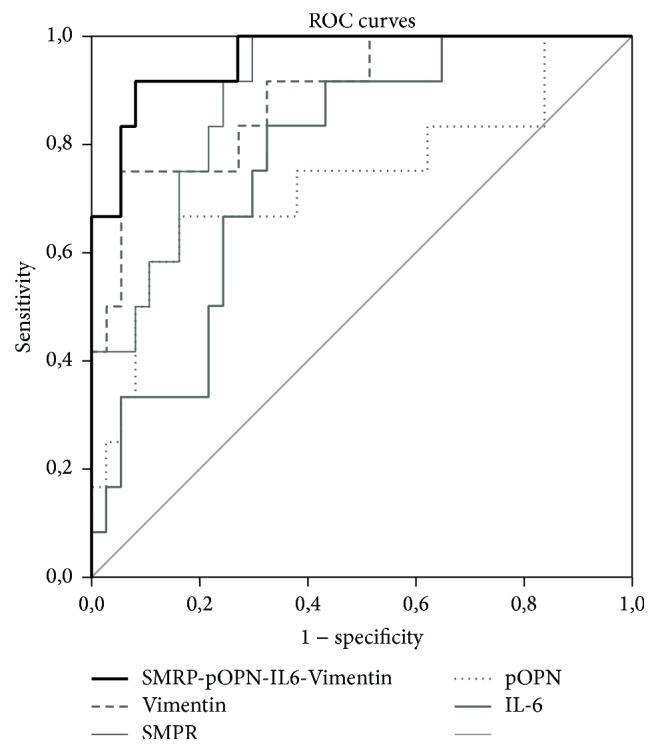
Biomarkers combinations were graphically shown.

THSP2 was excluded as diagnostic marker because of its low AUC value. The other markers were analyzed making several combinations, applying a model of logistic regression to determine the weight of each marker. As shown in Table 3 by the AUC values, the best markers were IL6 and pOPN, or SMRP and pOPN. Each of these combinations was tested with all other markers, one at time. The best three-marker combinations were IL6-pOPN-SMRP and IL6-pOPN-Desmin that reached an AUC value of 0.945 and 0.950, respectively. In the next step, all the other markers were added one at time to these two combinations. The resulting best four-marker combination was the one composed by SMRP, pOPN, IL6, and Vimentin, with an AUC value of 0.962 (CI of 0.910–1000), as show in Figure 1.

Using Youden Index, the best cut-off of this markers combination was 0.13, with a sensitivity of 100% and a specificity of 73%. When Desmin and HGF were added to this combination, at the best cut-off, the specificity reached 85.7%.

## 4. Discussion

Since the last decade, researches focused their attention on new diagnostic and/or prognostic biomarkers for MPM. This tumor is quite rare, but its incidence is increasing, due to the widespread asbestos use in the world.

In Italy, asbestos was banned in 1992, even if, in other parts of the world, such as countries from Asia, Africa, and South-America, asbestos is still employed in several industries and working processes.

The MPM is a tumor almost paradigmatic from the perspective of secondary prevention and early diagnosis. In fact, its long latency and the possibility to identifying a population at risk on the basis of previous exposure to asbestos make it a type of cancer on which to measure the importance of preventive strategies and health surveillance. Unfortunately, at present there are no established and unanimously accepted screening protocols. There is some debate on whether to perform regularly (and repeatedly) X-ray screening or better CT scan to the entire population of subjects with a past exposure to asbestos. In fact, it raises the ethical problem of exposure to nonnegligible doses of ionizing radiation.

In order to evolve a more reliable and less invasive protocol for the MPM, ideally, it would be desirable to identify biomarkers that allow an early diagnosis. These biomarkers could allow the identification of a grading of risk for MPM (thus allowing the modulation of the amount of radiological exams) and may increase the success of therapeutic approaches applied at an early stage of the disease. So, the identification of appropriate serum markers would lead to a revolution in the management of the surveillance of high-risk population and also in the possibility of diagnosis and treatment of patients.

To date, the most important biomarkers in MPM diagnosis and prognosis were SMRP and OPN, as shown by several authors in the research field [4, 6, 12, 13]. However, their use in the real clinical practice is limited because of an inadequate diagnostic accuracy. As in any other type of cancer, a biomarker combination could improve both sensitivity and specificity. In recent years, SMRP and/or pOPN were combined with other biomarkers, as CA125, CEA, and MPF (Megakaryocyte Potentiating Factor) [14–17]. All these combinations were performed using biomarkers already known for their employment in diagnosis of cancer different from MPM. Probably for this reason, biomarker combination did not reach appropriate values of sensitivity and specificity.

In this study, all the markers employed to make several combinations were specific for MPM diagnosis, deriving from very recent studies in mesothelioma field.

In 2014, Giusti et al. [10] performed a comparative proteomic analysis in MPM where some new proteins were suggested as potential biomarkers for MPM diagnosis. In particular the authors evidenced an altered expression of nuclear lamin and related filament proteins such as Vimentin and Desmin suggesting their ability to distinguish epithelioid mesothelioma from other lung malignancies with good values of sensibility and specificity.

Regarding genetics, very recently, Melaiu et al. [9] published a paper on genes differentially expressed in mesothelioma patients compared to healthy pleura, showing that several genes, including THSP2 and TIMP3, are differentially expressed between normal pleura and MPM. However, the expression of the mRNA usually shows a large overlap between healthy and malignant tissues leading to the idea that perhaps at mRNA level it is difficult to detect important differences enabling appropriate AUC for diagnostic discriminations.

After our recent studies using genomic and proteomic approaches, in the present study, specific ELISA were set up to validate whether the differentially expressed proteins were also detectable in the serum. Other markers, such as SMRP, pOPN, previously shown as diagnostic markers for MPM, and a panel of cytokines and grow factors (IL6, TNF-a, IL-5, Eotaxin, FGF-basic, PDGF-bb, VEGF, IP-10, CTACK, HGF, and SCGF-b) were studied. Antibodies or ELISA kit were purchased by commercial sources or produced when not commercially available, using commercial antigens or synthetic peptides corresponding to the immunogenic portions of the identified proteins.

First of all, all markers were explored to evaluate eventual confounding factors (sex and asbestos exposure) and it is of relevance to mention that no significant difference of biomarker serum levels was detectable between female/male and exposed/not exposed MPM patients.

Then, each marker was analyzed alone, comparing serum levels of MPM cases and subjects with a previous occupational exposure to asbestos. SMRP, pOPN, IL6, HGF, Desmin, IP10, Vimentin, and THSP2 were statistically different between the studied groups. Then, using a logistic regression method, these biomarkers were combined in several models, involving two, three, four, five, and six markers. As expected, an implementation of the biomarkers panel increased both sensitivity and specificity. Indeed, the combination of six biomarkers (SMRP-pOPN-IL6-Vimentin-Desmin-HGF) reached a sensitivity of 100% and a specificity of 85.7% at the best cut-off. Nevertheless, such result needs a careful interpretation considering the small number of patients examined. Although in terms of diagnostic efficiency these results are very interesting, we have to consider two major limitations. The first one is simply methodological and derives from the paucity of the cases sample analyzed. This is, more or less, a common weak-point of any research on MPM, suggesting the need for higher sized studies involving multiple research centers. The second limitation is of epidemiologic order since predictive values of biomarkers are negatively affected by very low incidence of disease. This study should be considered as preliminary. In fact, the number of recruited patients could not allow establishing a “discovery setting” (that allows the identification of biomarkers) and a “validation setting” (that allows the independent validation of the panel). However, we suggest that the combination of multiple markers could be very useful rather than the use of single markers in the diagnosis of MPM. Further studies are needed to validate these very promising results.

## Figures and Tables

**Table 1:** Concerning controls and MPM, age as median and standard deviation, percentage of males, smoking habit, and years of work as median and standard deviation.

	Age	Sex (males)	Smoking habit	Years of work	Asbestos exposure	Years asbestos exp
Controls (43)	58.7 ± 9.5	100%	30% no smokers 7% current smokers 63% ex-smokers	34.4 ± 6.5	100%	16.9 ± 8.5

MPM (27)	69.4 ± 7.9	78%	30% no smokers 11% current smokers 59% ex-smokers	32.7 ± 9.3	63%	19.5 ± 10.1^*∗*^

*∗*_ _ pertained to asbestos exposed patients.

**Table 2:** Biomarkers data were reported as mean ± SD (standard deviation) or median and 25°–75° percentile. The *p* value was considered significant when it was <0.5 (*∗*).

Marker	Unit	Subjects	Mean ± SD	Min	Max	*p* value
Eotaxin	pg/mL	Workers MPM	168.48 ± 74.02 165.70 ± 67.04	36.00 63.40	337.19 322.27	0.875
VEGF	pg/mL	Workers MPM	2133.19 ± 6066.87 459.25 ± 541.43	20.60 108.8	30140.00 3013.60	0.79
HGF	pg/mL	Workers MPM	337.49 ± 142.40 634.56 ± 436.26	155.83 83.25	832.56 1972.15	0.002^*∗*^
Lamin	ng/mL	Workers MPM	4875.15 ± 1723.54 4446.35 ± 1049.46	622.27 2642.23	9856.80 7305.91	0.287
Vimentin	ng/mL	Workers MPM	6.26 ± 2.34 8.06 ± 3.79	1.73 1.14	13.18 21.16	0.023^*∗*^

			Median	25° percentile	75° percentile	

Timp3	pg/mL	Workers MPM	29.60 27.20	18.41 16.87	41.04 36.43	0.6
THSP2	pg/mL	Workers MPM	21525 12370	15175 8395	26700 21220	0.023^*∗*^
SMRP	nM	Workers MPM	0.72 1.35	0.50 0.94	1.15 3.07	0.000^*∗*^
pOPN	ng/mL	Workers MPM	225.8 555.0	167.0 249.7	302.6 911.2	0.004^*∗*^
IL6	pg/mL	Workers MPM	11.4 22.81	7.4 14.85	14.8 39.26	0.000^*∗*^
TNFa	pg/mL	Workers MPM	28.58 32.65	19.33 22.03	37.91 42.41	0.655
IL5	pg/mL	Workers MPM	6.32 6.91	5.21 4.33	9.96 10.97	0.942
FGF basic	pg/mL	Workers MPM	74.85 82.71	51.72 53.90	104.18 115.11	0.534
PDGF-bb	pg/mL	Workers MPM	7084.53 5941.01	4095.57 2734.14	9098.03 9309.93	0.591
IP10	pg/mL	Workers MPM	941.15 1565.34	791.68 1122.36	1444.00 2421.65	0.002^*∗*^
CTAK	pg/mL	Workers MPM	578.34 566.95	461.75 450.95	780.41 896.65	0.947
SCGFb	pg/mL	Workers MPM	56887 47188	28627 37715	77644 61507	0.686
Desmin	ng/mL	Workers MPM	48.79 66.05	42.98 47.37	58.78 292.76	0.004^*∗*^
Fibulin	ng/mL	Workers MPM	372.94 385.55	251.31 155.87	430.12 533.33	0.983

**Table 3:** Roc curve analysis with AUC values of biomarker, alone or in combination.

AUC (IC 95%)		AUC of biomarker combination					
		SMRP	HGF	pOPN	Desmin	IP10	Vimentin
IL6	0.880 (0.800–0.961)	0.904	0.821	**0.910**	0.904	0.869	0.829
SMRP	0.795 (0.688–0.902)	—	0.866	**0.910**	0.837	0.837	0.862
HGF	0.767 (0.644–0.890)		—	0.881	0.813	0.804	0.792
pOPN	0.766 (0.597–0.934)			—	0.817	0.844	0.844
Desmin	0.739 (0.597–0.881)				—		0.731
IP10	0.719 (0.592–0.846)					—	0.774
Vimentin	0.685 (0.550–0.820)						—
THSP2	0.334 (0.192–0.476)						
